# Weighted arithmetic–geometric operator mean inequalities

**DOI:** 10.1186/s13660-018-1750-7

**Published:** 2018-07-03

**Authors:** Jianming Xue

**Affiliations:** Oxbridge College, Kunming University of Science and Technology, Kunming, P.R. China

**Keywords:** 47A63, 47A30, Positive linear map, Operator inequality, Weighted arithmetic operator mean, Weighted geometric operator mean

## Abstract

In this paper, we refine and generalize some weighted arithmetic–geometric operator mean inequalities due to Lin (Stud. Math. 215:187–194, 2013) and Zhang (Banach J. Math. Anal. 9:166–172, 2015) as follows: Let *A* and *B* be positive operators. If $0< m\le A\le{m}'<{M}'\le B\le M$ or $0< m\le B\le{m}'<{M}'\le A\le M$, then for a positive unital linear map Φ,

$$\begin{gathered} \Phi^{2} ( {A\nabla_{\alpha}B} ) \le \biggl[ { \frac{{K ( h )}}{{S ( {h^{\prime r} } )}}} \biggr]^{2} \Phi^{2} ( {A \sharp_{\alpha}B} ), \\ \Phi^{2} ( {A\nabla_{\alpha}B} ) \le \biggl[ { \frac{{K ( h )}}{{S ( {h^{\prime r} } )}}} \biggr]^{2} \bigl[ {\Phi ( A )\sharp_{\alpha}\Phi ( B )} \bigr]^{2}, \\ \Phi^{2p} ( {A\nabla_{\alpha}B} ) \le\frac{1}{{16}} \biggl[ { \frac{{K^{2} ( h ) ( {M^{2} + m^{2} } )^{2} }}{{S^{2} ( {h^{\prime r} } )M^{2} m^{2} }}} \biggr]^{p} \Phi^{2p} ( {A\sharp _{\alpha}B} ), \\ \Phi^{2p} ( {A\nabla_{\alpha}B} ) \le\frac{1}{{16}} \biggl[ { \frac{{K^{2} ( h ) ( {M^{2} + m^{2} } )^{2} }}{{S^{2} ( {h^{\prime r} } )M^{2} m^{2} }}} \biggr]^{p} \bigl[ {\Phi ( A )\sharp_{\alpha}\Phi ( B )} \bigr]^{2p},\end{gathered} $$

where $\alpha \in [ {0,1} ]$, $K ( h ) = \frac {{ ( {h + 1} )^{2} }}{{4h}}$, $S(h') = \frac{{h^{\prime \frac{1}{{h' - 1}}} }}{{e\log h^{\prime \frac{1}{{h' - 1}}} }} $, $h = \frac{M}{m} $, $h' = \frac{{M'}}{{m'}}$, $r = \min \{ {\alpha,1 - \alpha} \}$ and $p \ge2$.

## Introduction

Let $\mathcal{B(H)}$ be the $C^{*}$-algebra of all bounded linear operators on a Hilbert space $(\mathcal{H},\langle\cdot,\cdot\rangle)$ and *I* be the identity operator. $\Vert \cdot \Vert $ is the operator norm. $A\ge0$ ($A>0$) implies that *A* is a positive (strictly positive) operator. A linear map $\Phi:\mathcal{B(H)} \to\mathcal{B(K)}$ is called positive if $A\ge0$ implies $\Phi(A)\ge0$. It is said to be unital if $\Phi (I)=I$. For $A,B>0$, the *α*-weighted arithmetic mean and *α*-weighted geometric mean of *A* and *B* are defined, respectively, by

$$A\nabla_{\alpha}B = ( {1 - \alpha} )A + \alpha B,\qquad A\sharp _{\alpha}B = A^{\frac{1}{2}} \bigl( {A^{ - \frac{1}{2}} BA^{ - \frac {1}{2}} } \bigr)^{\alpha}A^{\frac{1}{2}}, $$

where $\alpha \in [ {0,1} ]$. When $\alpha = \frac{1}{2}$, we write $A\nabla B$ and $A\sharp B$ for brevity for $A\nabla_{\frac {1}{2}} B$ and $A\sharp_{\frac{1}{2}} B$, respectively.

Let $0 < m\le A,B \le M$, and Φ be a positive unital linear map. Tominaga [3] showed that the following operator inequality holds:

1.1 $$ \frac{{A + B}}{2} \le S ( h )A\sharp B, $$

where $S(h) = \frac{{h^{\frac{1}{{h - 1}}} }}{{e\log h^{\frac{1}{{h - 1}}} }}$ is called Specht’s radio and $h = \frac{M}{m}$. Indeed

1.2 $$ S(h) \le K(h) = \frac{{ ( {h + 1} )^{2} }}{{4h}} \le S^{2} (h)\quad (h \ge1) $$

was observed by Lin [1, (3.3)].

By (1.1) and (1.2), it is easy to obtain the following inequality:

1.3 $$ \Phi \biggl( {\frac{{A + B}}{2}} \biggr) \le K ( h )\Phi ( {A \sharp B} ). $$

Lin [1, Theorem 2.1] proved that (1.3) can be squared as follows:

1.4 $$ \Phi^{2} \biggl( {\frac{{A + B}}{2}} \biggr) \le K^{2} ( h )\Phi ^{2} ( {A\sharp B} ) $$

and

1.5 $$ \Phi^{2} \biggl( {\frac{{A + B}}{2}} \biggr) \le K^{2} ( h ) \bigl[ {\Phi ( A )\sharp\Phi ( B )} \bigr]^{2}. $$

Zhang [2] generalized (1.4) and (1.5) when $p \ge2$

1.6 $$ \Phi^{2p} \biggl( {\frac{{A + B}}{2}} \biggr) \le \frac{{ [ {K ( h ) ( {M^{2} + m^{2} } )} ]^{2p} }}{{16M^{2p} m^{2p} }}\Phi^{2p} ( {A\sharp B} ) $$

and

1.7 $$ \Phi^{2p} \biggl( {\frac{{A + B}}{2}} \biggr) \le \frac{{ [ {K ( h ) ( {M^{2} + m^{2} } )} ]^{2p} }}{{16M^{2p} m^{2p} }} \bigl[ {\Phi ( A )\sharp\Phi ( B )} \bigr]^{2p}. $$

A great number of results on operator inequalities have been given in the literature, for example, see [4–6] and the references therein.

In this paper, motivated by the aforementioned discussion, we extend (1.4)–(1.7) to the weighted arithmetic–geometric mean. In order to prove our results, we show a new operator weighted arithmetic–geometric mean inequality. Manipulating this operator inequality enables us to refine and generalize (1.4)–(1.7). Furthermore, a numerical example is given to demonstrate the effectiveness of the theoretical results.

## Main results

In this section, the main results of this paper will be given. To do this, the following lemmas are necessary.

### Lemma 1

([7])

*Let* $A,B>0$. *Then the following norm inequality holds*:

2.1 $$ \Vert {AB} \Vert \le\frac{1}{4} \Vert {A+B} \Vert ^{2}. $$

### Lemma 2

([8])

*Let* $A>0$. *Then for every positive unital linear map* Φ,

2.2 $$ \Phi \bigl(A^{-1} \bigr)\ge\Phi^{-1}(A). $$

### Lemma 3

([9])

*Let* $A,B>0$. *Then for* $1\le r<\infty$,

2.3 $$ \bigl\Vert {A^{r}+B^{r}} \bigr\Vert \le \bigl\Vert {(A+B)^{r}} \bigr\Vert . $$

### Lemma 4

([10])

*Let* $0< m\le A\le{m}'<{M}'\le B\le M$ *or* $0< m\le B\le{m}'<{M}'\le A\le M$. *Then for each* $\alpha \in [ {0,1} ]$,

2.4 $$ A\nabla_{\alpha}B \ge S \bigl( {h^{\prime r} } \bigr)A\sharp_{\alpha}B, $$

*where* $S(h') = \frac{{h^{\prime \frac{1}{{h' - 1}}} }}{{e\log h^{\prime \frac {1}{{h' - 1}}} }} $, $h' = \frac{{M'}}{{m'}}$ *and* $r = \min \{ {\alpha,1 - \alpha} \}$.

### Theorem 1

*Let* $0< m\le A\le{m}'<{M}'\le B\le M$ *or* $0< m\le B\le{m}'<{M}'\le A\le M$. *Then for each* $\alpha \in [ {0,1} ]$,

2.5 $$ A\nabla_{\alpha}B + MmS \bigl( {h^{\prime r} } \bigr) ( {A\sharp_{\alpha}B} )^{ - 1} \le M + m, $$

*where* $S(h') = \frac{{h^{\prime \frac{1}{{h' - 1}}} }}{{e\log h^{\prime \frac {1}{{h' - 1}}} }} $, $h' = \frac{{M'}}{{m'}}$ *and* $r = \min \{ {\alpha,1 - \alpha} \}$.

### Proof

Since

$$0 < m \le A \le M, $$

then

$$( {1 - \alpha} ) ( {M - A} ) ( {m - A} )A^{ - 1} \le0. $$

That is,

2.6 $$ ( {1 - \alpha} ) \bigl( {A + MmA^{ - 1} } \bigr) \le ( {1 - \alpha} ) ( {M + m} ). $$

Similarly, we get

2.7 $$ \alpha \bigl( {B + MmB^{ - 1} } \bigr) \le\alpha ( {M + m} ). $$

Summing up inequalities (2.6) and (2.7), we get

$$A\nabla_{\alpha}B + MmA^{ - 1} \nabla_{\alpha}B^{ - 1} \le M + m. $$

By $( {A\sharp_{\alpha}B} )^{ - 1} = A^{ - 1} \sharp _{\alpha}B^{ - 1} $ and (2.4), we have

$$\begin{aligned} A\nabla_{\alpha}B + MmS \bigl( {h^{\prime r} } \bigr) ( {A\sharp _{\alpha}B} )^{ - 1} &\le A\nabla_{\alpha}B + MmA^{ - 1} \nabla_{\alpha}B^{ - 1} \\ &\le M + m. \end{aligned} $$

This completes the proof. □

### Theorem 2

*Let* Φ *be a positive unital linear map and let* *A* *and* *B* *be positive operators*. *If* $0< m\le A\le{m}'<{M}'\le B\le M$ *or* $0< m\le B\le{m}'<{M}'\le A\le M$, *then for each* $\alpha \in [ {0,1} ]$,

2.8 $$\begin{aligned}& \Phi^{2} ( {A\nabla_{\alpha}B} ) \le \biggl[ { \frac{{K ( h )}}{{S ( {h^{\prime r} } )}}} \biggr]^{2} \Phi^{2} ( {A \sharp_{\alpha}B} ), \end{aligned}$$

2.9 $$\begin{aligned}& \Phi^{2} ( {A\nabla_{\alpha}B} ) \le \biggl[ { \frac{{K ( h )}}{{S ( {h^{\prime r} } )}}} \biggr]^{2} \bigl[ {\Phi ( A )\sharp_{\alpha}\Phi ( B )} \bigr]^{2}, \end{aligned}$$

*where* $K ( h ) = \frac{{ ( {h + 1} )^{2} }}{{4h}}$, $S(h') = \frac{{h^{\prime \frac{1}{{h' - 1}}} }}{{e\log h^{\prime \frac{1}{{h' - 1}}} }} $, $h = \frac{M}{m} $, $h' = \frac{{M'}}{{m'}}$ *and* $r = \min \{ {\alpha,1 - \alpha} \}$.

### Proof

Inequality (2.8) is equivalent to

$$\bigl\Vert {\Phi ( {A\nabla_{\alpha}B} )\Phi^{ - 1} ( {A \sharp_{\alpha}B} )} \bigr\Vert \le\frac{{K ( h )}}{{S ( {h^{\prime r} } )}}. $$

By (2.1), (2.2) and (2.5), we have

$$\begin{aligned} \bigl\Vert {\Phi ( {A\nabla_{\alpha}B} )MmS \bigl( {h^{\prime r} } \bigr)\Phi^{ - 1} ( {A \sharp_{\alpha}B} )} \bigr\Vert &\le \frac{1}{4} \bigl\Vert { \Phi ( {A\nabla_{\alpha}B} ) + MmS \bigl( {h^{\prime r} } \bigr)\Phi^{ - 1} ( {A\sharp_{\alpha}B} )} \bigr\Vert ^{2} \\ &\le \frac{1}{4} \bigl\Vert {\Phi ( {A\nabla_{\alpha}B} ) + MmS \bigl( {h^{\prime r} } \bigr)\Phi \bigl[ { ( {A \sharp_{\alpha}B} )^{ - 1} } \bigr]} \bigr\Vert ^{2} \\ &= \frac{1}{4} \bigl\Vert {\Phi \bigl[ { ( {A\nabla_{\alpha}B} ) + MmS \bigl( {h^{\prime r} } \bigr) ( {A\sharp_{\alpha}B} )^{ - 1} } \bigr]} \bigr\Vert ^{2} \\ &\le \frac{1}{4} \bigl\Vert {\Phi ( {M + m} )} \bigr\Vert ^{2} \\ &= \frac{1}{4} ( {M + m} )^{2}. \end{aligned} $$

That is,

$$\bigl\Vert {\Phi ( {A\nabla_{\alpha}B} )\Phi^{ - 1} ( {A \sharp_{\alpha}B} )} \bigr\Vert \le\frac{{ ( {M + m} )^{2} }}{{4MmS ( {h^{\prime r} } )}} = \frac{{K ( h )}}{{S ( {h^{\prime r} } )}}. $$

Thus, (2.8) holds.

Inequality (2.9) is equivalent to

$$\bigl\Vert {\Phi ( {A\nabla_{\alpha}B} ) \bigl[ {\Phi ( A ) \sharp_{\alpha}\Phi ( B )} \bigr]^{ - 1} } \bigr\Vert \le \frac{{K ( h )}}{{S ( {h^{\prime r} } )}}. $$

By (2.1) and (2.5), we have

$$\begin{gathered} \bigl\Vert {\Phi ( {A\nabla_{\alpha}B} )MmS \bigl( {h^{\prime r} } \bigr) \bigl[ {\Phi ( A ) \sharp_{\alpha}\Phi ( B )} \bigr]^{ - 1} } \bigr\Vert \\ \quad\le\frac{1}{4} \bigl\Vert {\Phi ( {A\nabla_{\alpha}B} ) + MmS \bigl( {h^{\prime r} } \bigr) \bigl[ {\Phi ( A ) \sharp_{\alpha}\Phi ( B )} \bigr]^{ - 1} } \bigr\Vert ^{2} \\ \quad= \frac{1}{4} \bigl\Vert {\Phi ( A )\nabla_{\alpha}\Phi ( B ) + MmS \bigl( {h^{\prime r} } \bigr) \bigl[ {\Phi ( A ) \sharp_{\alpha}\Phi ( B )} \bigr]^{ - 1} } \bigr\Vert ^{2} \\ \quad\le\frac{1}{4} ( {M + m} )^{2}. \end{gathered} $$

That is,

$$\bigl\Vert {\Phi ( {A\nabla_{\alpha}B} ) \bigl[ {\Phi ( A ) \sharp_{\alpha}\Phi ( B )} \bigr]^{ - 1} } \bigr\Vert \le \frac{{K ( h )}}{{S ( {h^{\prime r} } )}}. $$

Thus, (2.9) holds.

This completes the proof. □

### Theorem 3

*Let* Φ *be a positive unital linear map and let* *A* *and* *B* *be positive operators*. *If* $0< m\le A\le{m}'<{M}'\le B\le M$ *or* $0< m\le B\le{m}'<{M}'\le A\le M$ *and* $2 \le p < \infty$, *then for each* $\alpha \in [ {0,1} ]$,

2.10 $$\begin{aligned}& \Phi^{2p} ( {A\nabla_{\alpha}B} ) \le \frac{1}{{16}} \biggl[ {\frac{{K^{2} ( h ) ( {M^{2} + m^{2} } )^{2} }}{{S^{2} ( {h^{\prime r} } )M^{2} m^{2} }}} \biggr]^{p} \Phi^{2p} ( {A\sharp _{\alpha}B} ), \end{aligned}$$

2.11 $$\begin{aligned}& \Phi^{2p} ( {A\nabla_{\alpha}B} ) \le \frac{1}{{16}} \biggl[ {\frac{{K^{2} ( h ) ( {M^{2} + m^{2} } )^{2} }}{{S^{2} ( {h^{\prime r} } )M^{2} m^{2} }}} \biggr]^{p} \bigl[ {\Phi ( A )\sharp_{\alpha}\Phi ( B )} \bigr]^{2p}, \end{aligned}$$

*where* $K ( h ) = \frac{{ ( {h + 1} )^{2} }}{{4h}}$, $S(h') = \frac{{h^{\prime \frac{1}{{h' - 1}}} }}{{e\log h^{\prime \frac{1}{{h' - 1}}} }} $, $h = \frac{M}{m} $, $h' = \frac{{M'}}{{m'}}$ *and* $r = \min \{ {\alpha,1 - \alpha} \}$.

### Proof

By (2.8), we have

2.12 $$ \Phi^{ - 2} ( {A\sharp_{\alpha}B} ) \le L^{2} \Phi^{ - 2} ( {A\nabla_{\alpha}B} ), $$

where $L = \frac{{K(h)}}{{S(h^{\prime r} )}}$.

Inequality (2.10) is equivalent to

$$\bigl\Vert {\Phi^{p} ( {A\nabla_{\alpha}B} ) \Phi^{ - p} ( {A\sharp_{\alpha}B} )} \bigr\Vert \le \frac{1}{4} \biggl[ {\frac {{K^{2} ( h ) ( {M^{2} + m^{2} } )^{2} }}{{S^{2} ( {h^{\prime r} } )M^{2} m^{2} }}} \biggr]^{\frac{p}{2}}. $$

By (2.1), (2.3) and (2.12), we have

$$\begin{aligned}& \bigl\Vert {\Phi^{p} ( {A \nabla_{\alpha}B} )M^{p} m^{p} \Phi^{ - p} ( {A \sharp_{\alpha}B} )} \bigr\Vert \\& \quad\le\frac{1}{4} \biggl\Vert {L^{\frac{p}{2}} \Phi^{p} ( {A\nabla _{\alpha}B} ) + \biggl( {\frac{{M^{2} m^{2} }}{L}} \biggr)^{\frac {p}{2}} \Phi^{ - p} ( {A\sharp_{\alpha}B} )} \biggr\Vert ^{2} \\& \quad\le\frac{1}{4} \biggl\Vert {L\Phi^{2} ( {A \nabla_{\alpha}B} ) + \frac{{M^{2} m^{2} }}{L}\Phi^{ - 2} ( {A \sharp_{\alpha}B} )} \biggr\Vert ^{p} \\& \quad\le \frac{1}{4} \bigl\Vert {L\Phi^{2} ( {A \nabla_{\alpha}B} ) + LM^{2} m^{2} \Phi^{ - 2} ( {A\nabla_{\alpha}B} )} \bigr\Vert ^{p} \\& \quad\le\frac{1}{4} \bigl[ {L \bigl( {M^{2} + m^{2} } \bigr)} \bigr]^{p}. \end{aligned}$$

That is,

$$\bigl\Vert {\Phi^{p} ( {A\nabla_{\alpha}B} ) \Phi^{ - p} ( {A\sharp_{\alpha}B} )} \bigr\Vert \le \frac{1}{4} \biggl[ {\frac {{L ( {M^{2} + m^{2} } )}}{{Mm}}} \biggr]^{p} = \frac{1}{4} \biggl[ {\frac{{K^{2} ( h ) ( {M^{2} + m^{2} } )^{2} }}{{S^{2} ( {h^{\prime r} } )M^{2} m^{2} }}} \biggr]^{\frac{p}{2}}. $$

Thus, (2.10) holds.

Similarly, (2.11) holds by inequality (2.9).

This completes the proof. □

### Remark 1

When $\alpha = \frac{1}{2}$, because of $\frac {{K ( h )}}{{S ( {\sqrt{h'} } )}} < K ( h )$, inequalities (2.8), (2.9), (2.10) and (2.11) are sharper than (1.4), (1.5), (1.6) and (1.7), respectively.

In what follows, when $\alpha=\frac{1}{2}$, we present an example showing that inequalities (2.8)–(2.11) are sharper than (1.4)–(1.7), respectively.

### Example 1

Take $A = \bigl[ { {\scriptsize\begin{matrix}{} {\frac{2}{3}} & 0 \cr 0 & {\frac{5}{7}} \end{matrix}} } \bigr] $ and $B = \bigl[ { {\scriptsize\begin{matrix}{} {\frac{10}{3}} & 0 \cr 0 & {\frac{23}{7}} \end{matrix}} } \bigr]$. We find $\frac{1}{2} < A < \frac{3}{4} < 3 < B < 4$. A calculation shows $\frac{{K(8)}}{{S(2)}} \approx2.3847 < K(8) \approx2.5313$.

## Conclusions

In this paper, we have presented some new weighted arithmetic–geometric operator mean inequalities. These inequalities are refinements and generalizations of some corresponding results of [1, 2].

## References

[1] Lin M. (2013). Squaring a reverse AM-GM inequality. Stud. Math..

[2] Zhang P. (2015). More operator inequalities for positive linear maps. Banach J. Math. Anal..

[3] Tominaga M. (2002). Specht’s ratio in the Young inequality. Sci. Math. Jpn..

[4] Xue J. (2017). Some refinements of operator reverse AM-GM mean inequalities. J. Inequal. Appl..

[5] Zou L. (2017). An arithmetic-geometric mean inequality for singular values and its applications. Linear Algebra Appl..

[6] Hu X. (2012). Some inequalities for unitarily invariant norms. J. Math. Inequal..

[7] Bhatia R., Kittaneh F. (2000). Notes on matrix arithmetic-geometric mean inequalities. Linear Algebra Appl..

[8] Bhatia R. (2007). Positive Definite Matrices.

[9] Ando T., Zhan X. (1999). Norm inequalities related to operator monotone functions. Math. Ann..

[10] Zuo H., Shi G., Fujii M. (2011). Refined Young inequality with Kantorovich constant. J. Math. Inequal..

